# Individuation and holistic processing of faces in rhesus monkeys

**DOI:** 10.1098/rspb.2007.0477

**Published:** 2007-06-26

**Authors:** Christoph D Dahl, Nikos K Logothetis, Kari L Hoffman

**Affiliations:** Max Planck Institute for Biological CyberneticsTübingen 72012, Germany

**Keywords:** face perception, scan path, habituation, dishabituation, preferential looking, inferotemporal cortex

## Abstract

Despite considerable evidence that neural activity in monkeys reflects various aspects of face perception, relatively little is known about monkeys' face processing abilities. Two characteristics of face processing observed in humans are a *subordinate-level entry point*, here, the default recognition of faces at the subordinate, rather than basic, level of categorization, and *holistic effects*, i.e. perception of facial displays as an integrated whole. The present study used an adaptation paradigm to test whether untrained rhesus macaques (*Macaca mulatta*) display these hallmarks of face processing. In experiments 1 and 2, macaques showed greater rebound from adaptation to conspecific faces than to other animals at the individual or subordinate level. In experiment 3, exchanging only the bottom half of a monkey face produced greater rebound in aligned than in misaligned composites, indicating that for normal, aligned faces, the new bottom half may have influenced the perception of the whole face. Scan path analysis supported this assertion: during rebound, fixation to the *unchanged* eye region was renewed, but only for aligned stimuli. These experiments show that macaques naturally display the distinguishing characteristics of face processing seen in humans and provide the first clear demonstration that holistic information guides scan paths for conspecific faces.

## 1. Introduction

The question of whether and to what extent faces are processed differently when compared with non-face objects has been a major focus of research in humans. Converging evidence indicates that one key distinction is the holistic processing of faces. For example, parts presented in the context of a whole face are recognized better than when presented in isolation (Tanaka & Farah 1993). Moreover, when faces are split into top and bottom halves, observers are influenced by the half that they were supposed to ignore, but only when the halves are aligned (Young *et al*. 1987; Hole 1994). The facilitation of performance for whole faces, as well as the relative inability to selectively attend to (or ignore) face parts, indicates that the face is normally processed as a single, indivisible entity, i.e. faces are processed *holistically*.

The recognition impairments that occur when faces are presented upside down (the ‘face inversion effect’; Yin 1969; Valentine 1988; Valentine & Bruce 1988) have also been taken as evidence for holistic processing (Maurer *et al*. 2002). Yet the effect is also seen when detecting changes in the configuration of facial features, such as eye spacing, even when these face parts are presented in isolation (Leder & Bruce 2000; Leder *et al*. 2001). The impairments in discriminating such second-order relational (or configural) manipulations for inverted faces are quite robust (e.g. Phillips & Rawles 1979; Bartlett & Searcy 1993; Rhodes *et al*. 1993); nevertheless, some experiments suggest that the face inversion effect is *not* the result of qualitatively different processing strategies between upright and inverted faces (Nachson & Shechory 2002; Rakover 2002; Sekuler *et al*. 2004). Instead, the effect may be due to increased difficulty in processing many aspects of the less familiar inverted orientation (Bradshaw & Wallace 1971; Valentine & Bruce 1986; Valentine 1988). Moreover, different processing strategies may emerge as an artefact of the experimental design (Riesenhuber *et al*. 2004). In sum, it is not clear whether the face inversion paradigm is well suited to identify and compare distinct face processing strategies across species.

Another distinguishing feature of face processing is the default, or entry point, level of categorization. Whereas most non-face objects are identified at the basic level, faces are identified subordinate to the basic level, at the level of the individual. For example, an image of a dog would be labelled ‘dog’ (basic) over ‘Rover’ (individual), yet a face is often labelled, for example, ‘Elvis’ over ‘face’ (Rosch *et al*. 1976; Jolicoeur *et al*. 1984; Tanaka 2001). The subordinate-level entry point for objects of expertise, such as faces, has been observed in numerous studies using varied techniques (Tanaka & Taylor 1991; Gauthier & Tarr 1997; Johnson & Mervis 1997; Tanaka 2001; Tanaka *et al*. 2005; but see Grill-Spector & Kanwisher 2005). The processing mechanism(s) enabling a subordinate entry point for faces is not clearly specified—it could be the idiosyncratic features of an individual's face (featural), the unique spatial relationships of facial features (configural) or a unitary ‘template’ of the face of that individual (holistic). As such, the subordinate-level entry point marks a second, independent, aspect of face processing not typically seen for non-face objects.

These behavioural results have formed a foundation on which to explore the neural basis of face perception. Some of the most direct evidence for selective representation of faces in the brain arises from the electrophysiological studies of the temporal lobe of macaques (Gross *et al*. 1972; Bruce *et al*. 1981; Perrett *et al*. 1982; Desimone *et al*. 1984). Despite the numerous studies of face coding in the monkey brain, there has been relatively little research on the face processing abilities of the monkeys themselves, particularly in relation to the behavioural research in humans.

Our understanding of face perception in the monkey comes almost exclusively from the study of the face inversion effect, with mixed results. Macaques choose to look longer at upright than inverted images of conspecifics (Tomonaga 1994; Guo *et al*. 2003), though this does not necessarily indicate that they would show discrimination impairments for inverted compared with upright faces, i.e. a face inversion effect. Unfortunately, most of these studies used explicit reinforcement for some type of discrimination (Rosenfeld & Van Hoesen 1979; Bruce 1982; Overman & Doty 1982; Perrett *et al*. 1988; Keating & Keating 1993; Wright & Roberts 1996; Parr *et al*. 1999). When tested, these protocols appeared to produce systematically altered or idiosyncratic response strategies, making it difficult to disentangle what monkeys are capable of learning from what they would do under natural circumstances (Perrett *et al*. 1988; Keating & Keating 1993). Moreover, the face inversion effect in humans may not be the result of holistic processing applied preferentially to upright faces; it may be merely the result of less experience discriminating upside down faces (Sekuler *et al*. 2004). Thus, even if untrained macaques showed robust face inversion effects, it would not be clear that this was the result of the key attributes of face processing seen in humans.

Few studies have tried to address a second hallmark of face perception in monkeys, namely the entry point of categorization. Although macaques can discriminate the faces of other monkeys (Bruce 1982; Pascalis & Bachevalier 1998), it is not clear how this relates to their ability to discriminate faces at the basic level (compared with objects), nor to their discrimination of subordinate-level objects. One landmark study used an adaptation, or dishabituation, paradigm to reveal that the rebound in looking time following changes in a monkey's identity was as great as the rebound following a cross-species (or basic-level) change (Humphrey 1974). In contrast, changes of identity within other domestic animal categories produced no significant rebound; only the basic-level, cross-species changes were significant. Thus, monkeys that had not been explicitly trained to differentiate images nevertheless displayed a subordinate-level entry point which was selective to conspecifics. Although hierarchical categorization has been observed in tamarins (Neiworth *et al*. 2004) and Sulawesi macaques (Fujita *et al*. 1997), these studies presented pictures of the entire monkey, thus it is not clear whether a subordinate-level entry point would be observed for faces presented in isolation.

Taken together, there have been indications that adult monkeys naturally individuate other monkeys (Humphrey 1974), and that monkeys, like chimpanzees, are *able* to process a configuration of facial features (Overman & Doty 1982; Perrett *et al*. 1988; Parr *et al*. 1999), yet no evidence to date shows that monkeys' natural face processing abilities involve holistic processing or a subordinate-level entry point.

Here, we measure the untrained responses of macaques (*Macaca mulatta*) to address the following questions: (i) Do macaques differentiate conspecific faces better than other subordinate-level stimuli (e.g. dogs, birds or another monkey species)? (ii) Is their face perception characterized by holistic processing?

In experiment 1, the entry point of face categorization is measured using an adaptation task (figure 1*a*) modelled after that described by Humphrey (1974).To the extent that monkeys individuate conspecific faces, greater rebound from adaptation is expected for trials of faces at the subordinate, relative to basic, level than for trials of other animals at the subordinate, relative to basic, level. In addition, configural information processing was investigated by manipulating the inter-ocular distance of monkey face images. If perceived similarity of faces is dependent on featural configuration, we should observe more rebound for faces that have undergone a configural manipulation and are rotated in plane than for the identical face merely rotated in plane (figure 1*a*). Furthermore, as tested in experiment 2, if individuation of faces is species specific, then the relative subordinate-level rebound should be greater for macaque faces than for another species' faces (e.g. marmosets).

In experiment 3, holistic processing of faces was tested by adapting the test monkeys to composites of either vertically aligned or misaligned faces (figure 1*c*). If the aligned face is processed holistically, then presentation of a new bottom half should cause greater rebound in the aligned than in the misaligned condition, as though a face with a new bottom half is perceived as a whole new face. In this design, the tendency for gaze to be directed to novel parts of an image conflicts with monkeys' tendency to fixate the eye region, particularly when viewing new faces. Scan paths to the eye region during rebound periods will be compared in aligned and misaligned conditions, to determine whether scan patterns in the aligned condition also rebound, resembling those seen when viewing a new face.

## 2. Material and methods

### (a) Subjects

Five male Rhesus monkeys (3–10 years old, 5–13 kg) were socially housed with one to three monkeys in the same cage, in a colony of approximately 30 monkeys. Each monkey was implanted under sterile conditions with a custom-designed, form-fitting titanium head post (Logothetis *et al*. 2002). All monkeys were habituated to head restraint prior to testing and were naive to the stimuli used in the current experiments.

### (b) Stimuli

In experiment 1, digital colour pictures of three object categories were used: birds; dogs; and rhesus monkey faces. Five dog and five bird images were obtained from Animal Picture Archive (www.animalpicturesarchive.com) and 16 monkey images (one image of each of 16 monkeys) were obtained from the California National Primate Research Center in Davis, California provided by Dr Katalin Gothard, University of Arizona (Gothard *et al*. 2004). Each category exemplar was extracted from its original background, normalized for luminance and placed on a mid-grey background at a resolution of 300×300 pixels (figure 1*a*). Monkey faces were aligned approximately 15° from vertical, allowing the same image to be presented as a 30° in-plane rotated image (monkey) or as a mirror image (dog and bird), as a means of varying the stimulus without a corresponding change in the image content (Humphrey 1974). For configural trials, the inter-ocular distance of monkey face images was manipulated using WinMorph v. 3.01 software (by Satish Kumar). Before tilting the picture, eyes were displaced horizontally by 5–10 pixels on both sides; intervening points were stretched proportionately, maintaining the nearest neighbour relationship of all pixels. This displacement kept the inter-ocular distance to within 2 s.d. of the mean, taken from nine monkeys in our colony, (colony mean (s.d.): 37 mm (3.62); range 32.5–39.5 mm; image mean (s.d.): 36.1 mm (3.55) and 37.3 mm (4.05) before and after manipulation, respectively, assuming an average head size). In addition to the images, a mid-grey blank square of the same size was created, as well as a grey outline demarcating the frame of 13.3°×13.3° visual angle.

Experiment 2 used 10 new macaque faces from the same face database and 10 marmoset faces. Nine of the marmoset faces were digitally captured from a laboratory colony (Nikon D70 digital camera; Panasonic NV-DS15 digital video camera) and one was downloaded from a web source (Raimond Spekking/Wikipedia) under the GNU Free Documentation License (http://www.gnu.org/copyleft/fdl.html). All 20 images were then processed as described for experiment 1.

In experiment 3, pictures of composite monkey faces were created by combining the top half of one face with the bottom half of another face. Stimuli subtended a visual angle of 20°×13.3°, with a black line running horizontally across the centre of the image, to maintain similar image discontinuities across conditions (figure 1*c*). Each of the eight top halves was combined with three bottom halves for a total of 24 composites. Each composite was presented in aligned and misaligned configurations. Aligned stimuli contained a centrally positioned composite face; misaligned stimuli presented the top half shifted 75 pixels to the left side and the bottom half shifted 75 pixels to the right side (figure 1*c*). As in experiment 1, a grey blank square and a grey outline of a matching size were created (450×300 pixels resolution, visual angle of 20.0°). An additional four misaligned stimuli (two unique top halves) were created with the eye regions centred and the lower half offset to the right of the eyes by the same 150 pixels as the original misaligned stimuli, for a total image size of 600×300 pixels. The background of four aligned stimuli (two unique top halves) was then modified to be size matched.

### (c) Adaptation procedure

During the experiment, the monkey was head restrained and seated in a primate chair inside a darkened booth in front of a 21-in. colour monitor (Digital, model: VRC21-HA) at a distance of approximately 94 cm. Eye movements of the monkeys were measured by an iView infrared eye tracking system (SensoMotoric Instruments (SMI), Teltow/Berlin, Germany) sampled at 200 Hz. Visual stimuli were presented with custom-written software, controlled and recorded with a QNX real-time operating system (QNX Software Systems, Canada).

The monkey initiated a trial by placing its gaze inside the grey outline. Either a blank grey square or an image would appear for as long as gaze was maintained within the image frame, up to a maximum of 5 s. The grey outline would remain after the stimulus was removed, to indicate the image boundary. When the monkey's gaze entered the gaze boundary anew, the alternate stimulus (image or blank square) was displayed (see movies in the electronic supplementary material). The trial was completed after a cumulative within-frame looking time of 20 s, regardless of the distribution across blank square and image stimuli. The monkey was provided juice during an intertrial delay of 5 s, irrespective of task behaviours, similar to the procedure described by Humphrey (1974).

Viewing preference was measured as the proportion of time spent looking at the picture to the total time looking at the picture and the blank square combined. Initially, monkeys show a preference for the picture, but over time, this preference diminishes or adapts. This preference can be re-established by presenting a new picture, and its magnitude varies based on the monkey's perception of how different, or novel, the new picture is to the adapted picture. Since the pre-exposure to one picture influences the preference for another picture (Humphrey 1974), we systematically varied the image category of each subsequent trial; thus, each trial (except for the first) provided a rebound from the adaptation of the trial preceding it.

#### (i) Experiment 1

Novel ‘rebound’ trials of monkey faces fell into one of four types (figure 1*a*): those preceded by (i) an animal picture (*dog* or *bird*) (‘basic’), (ii) a picture of a different macaque face (‘subordinate’), (iii) a configurally modified image of the same macaque, also rotated in plane (configural), or (iv) the rotated image of the same macaque face picture (‘same’). Novel trials of animal pictures fell into three of the four types: those preceded by (i) a macaque face picture (basic), (ii) a different, within-class dog or bird picture (subordinate), and (iii) the rotated image of the same animal picture (same).

Our primary objective was to present all conditions within one session, with a minimal number of trials. This was accomplished by presenting eight images (seven conditions) each consisting of 20 s of cumulative looking time at the image and blank square. This yielded exactly one trial per condition, such that one trial's novel image was the ‘habituation’ to the next trial. Six of these daily sessions were completed for each of the four monkeys. Within the constraints imposed by the trial types, we balanced the order of occurrence for conditions (e.g. subordinate-face trials preceded across-face trials in three out of six sessions) and made sure that both face and animal stimulus classes had trials in both early and late halves of the session. An example for one session would be: subordinate face; same face; basic dog; same dog; subordinate dog; basic face; and configural face.

Two sessions of one monkey were excluded due to drowsiness, and two sessions of a second monkey were excluded due to technical problems with data acquisition and display, leaving a total of 20 trials of each condition across monkeys and sessions.

#### (ii) Experiment 2

Four monkeys were tested: three that had completed experiment 1 and one previously untested monkey. The experimental design was identical to that of experiment 1, but stimuli consisted of unfamiliar macaque and marmoset faces, and no configural manipulation was presented (figure 1*b*). To minimize the sheer novelty of seeing a marmoset, videos of one marmoset and one rhesus monkey were played to the test monkeys before beginning the first session. Two of the test monkeys completed four sessions; the remaining two monkeys completed eight sessions, for a total of 24 sessions (hence 24 samples per condition) in the pooled data. The first session of one monkey was discarded due to restlessness; an additional two sessions in two monkeys were unusable due to technical problems; however, stimuli from these sessions were repeated successfully in later sessions, thus the total number of sessions analysed was kept constant at 24.

#### (iii) Experiment 3

The basic adaptation procedure was identical to that of the other experiments. In this experiment, however, a rebound trial could consist of an aligned monkey face preceded by another aligned monkey face or of a misaligned monkey face preceded by another misaligned monkey face (figure 1*c*). Each session contained the three aligned composites from one top half, as well as the three misaligned composites from another top half. Thus, although the consecutively presented composite images reflect featural (mouth) and configural (eye-to-mouth distance) changes, these changes are consistent across conditions. What differs is whether the changes are made to an intact (aligned) or disrupted (misaligned) face.

All four test monkeys from experiment 1 completed seven daily sessions comprising two rebound trials for aligned composites and two for misaligned composites pooled across sessions and monkeys, for a total of 56 trials per condition (figure 1*c*). The order of conditions was counterbalanced across eight sessions, producing a total of 16 aligned and 16 misaligned rebound trials for each monkey.

### (d) Data analysis

Viewing preference is measured as the proportion of time spent looking at the picture to the total time looking at the picture and the blank square combined. The response time window during rebound trials (i.e. ‘rebound window’) used for subsequent analysis of the category entry point and aligned/misaligned trials was 8 s. After this time point, responses across conditions began to show adaptation. For the more subtle image manipulations, the rebound window was reduced: a 3–6 s window was chosen for configural trials and the first 2 s of the trial were used for the macaque and marmoset faces used in experiment 2. Unless otherwise indicated, data were pooled across sessions and monkeys, and statistical analyses were performed using two-sample *t*-tests.

In experiment 3, fixation durations in the eye region were divided by the total time looking at the image during the rebound window. The eye region was defined as a rectangular area, in which upper and vertical sides were aligned to the monkey's eyebrows and hairline, respectively. The lower side of the rectangle was adjacent to the black line separating top and bottom halves. Any consecutive eye positions that differed by less than 0.3° of visual angle were considered fixations.

The fixation durations for the background region in the top half were calculated as the proportion of time spent looking at the upper half of the background to the total time looking at the picture. Data were pooled across sessions and trials (adaptation and novel) and statistically compared between aligned and misaligned. For display purposes only, densities were calculated at a resolution of 0.125° of visual angle and spatially smoothed by a Gaussian kernel with a standard deviation of 0.3125° of visual angle (figure 4).

## 3. Results

To address whether monkeys individuate conspecific faces, the principal effect of category entry point was analysed. Rebound from adaptation, also referred to as dishabituation, was used to measure perceived dissimilarity of stimuli varying at the basic- and subordinate-level category for monkey faces versus animals (figure 1*a*). First, rebound to a new monkey face following adaptation to another monkey face (subordinate) was compared with rebound in response to a rotation of the same face (same). Rebound is measured as the preference ratio, or the proportion of time spent viewing the new image versus an alternating blank stimulus (§2). The preference ratio of subordinate trials was significantly greater (*t*(38)=3.58; *p*<0.001) than that of same trials. Critically, basic and subordinate trials of monkey faces were contrasted to basic and subordinate trials of animals, to differentiate the category entry point across the two stimulus classes. If faces, but not animals, are individuated, then the perceived difference between two animals would be small compared to the difference across face–animal pairs, whereas the perceived difference between two monkey faces would be much closer to the across-category difference. A mixed-factor analysis of variance (ANOVA), evaluating effects of stimulus class (monkeys versus animals) and categorization level (basic versus subordinate) on preference ratio showed a significant interaction of stimulus classes and stimulus type (figure 2*a*; *F*(1,19)=7.03, *p*<0.05), indicating that monkey faces, but not animals, were perceived at the subordinate level. Similarly, when expressing the subordinate-level rebound as a fraction of the basic-level rebound, we expect non-face objects to produce a lower ratio than faces. Indeed, the ratios from each of the monkeys showed a greater deficit between subordinate and basic rebound for animals than for faces (*paired t*(3)=3.31, *p*<0.05).

To determine whether face perception in monkeys is sensitive to configurations of face parts, rebound for configural and same trials was compared. A two-sample *t*-test revealed that configural trials (*t*(38)=2.18; *p*<0.05) showed greater preference ratios overall than same trials (figure 2*a*). One of the four monkeys did not show this effect, thus sensitivity to inter-ocular spacing may not be a ubiquitous or robust feature of face processing in macaques.

In experiment 2, the specificity of increased rebound for faces was tested by presenting faces of marmosets and rhesus macaques. The results were nearly identical to those of experiment 1: the mixed-factor ANOVA revealed a significant interaction between species and category level (figure 2*b*; *F*(1,23)=6.52, *p*<0.05); the rebound for macaque faces in subordinate-level trials relative to basic-level trials is greater than that of marmoset faces across the same conditions. This indicates a greater perceived difference of macaque faces than of marmoset faces.

In experiment 3, we tested whether conspecific faces are processed holistically. Rebound for new aligned trials was contrasted with that of new misaligned trials. There was no significant difference in preference ratio to the aligned and misaligned trials during the adaptation phase. In contrast, when the lower half of each image was swapped, the preference ratio was greater in aligned than in misaligned trials (*t*(110)=2.03; *p*<0.05; aligned mean (s.e.m.)=0.54 (0.02); misaligned mean (s.e.m.)=0.49 (0.017); figure 3), consistent with holistic processing of the aligned stimuli. This effect was observed despite the overall lower preference ratio seen in one monkey across all conditions. Indeed, when comparing the mean response of each monkey for aligned and misaligned conditions, greater rebound from adaptation was observed for aligned than for misaligned trials (*paired t*(3)=7.48; *p*<0.01).

One alternate interpretation of these results is that change is more easily detected in parts that are closer to the salient eye region; thus, more rebound occurs for the *proximal* aligned bottom half than for the *distal* misaligned bottom half. This observation leads to two opposing predictions for scan paths. If proximity to the eye region increases the detectability of the bottom half, then the proportion of time spent viewing the eyes should decrease when a novel bottom half is presented in the aligned condition, and this decrement should exceed that of the (distal) misaligned trials. If, instead, the aligned face is perceived holistically, then the scan path during the aligned trial should show renewed fixations of the eye region, in contrast to decreasing fixations of the eye region in the misaligned trials. Consistent with the latter, holistic account, fixations of the eye region in the novel aligned trials showed no decrement from that of the original face presentation. In contrast, fixations of the eye region were reduced in novel misaligned trials relative to the original adaptation trial (see figure 4; movies in the electronic supplementary material; novel 1: *t*(54)=2.97, *p*<0.01; novel 2: *t*(54)=2.74, *p*<0.01; or both *p*<0.05, Bonferroni corrected for multiple comparisons). This deficit was seen *despite* lower fixation values in the misaligned compared with aligned adaptation condition (*t*(54)=1.92; *p*<0.05; figure 4).

Two effects appeared to underlie the initial difference in eye fixations. First, in misaligned trials, some fixations occurred immediately above the lower face half, where the eye region would naturally occur. The difference in eye region fixations across condition may have been attributable to these ‘offset’ fixations. To determine whether fixations to this region were non-random, excluding those to the existing face boundary, fixations in the total background region of the top half in the misaligned condition were compared with those in the aligned condition, and were found to be significantly greater in misaligned than in aligned conditions (*t*(166)=−2.37; *p*<0.01; figure 1 in the electronic supplementary material). This suggests that, in misaligned trials, the fixations to the novel bottom half may have been distributed between the bottom half and the adjacent regions in the top half, where an aligned eye region would fall. Second, the aligned trials contained centrally positioned eye regions, whereas the eyes in misaligned trials were shifted to the left side. To determine whether centrally presented eyes (i) altered the scan paths, and (ii) affected the rebound to a new lower half, we retested one of the monkeys on misaligned stimuli containing centred eyes. Relative to the original misaligned stimuli, the centred-eye misaligned images led to greater fixation of the eye region during the adaptation phase, but this did not prevent a shift to the lower half of the image in novel trials. In fact, the proportion of eye fixations in aligned trials exceeded those of misaligned trials even when measured *relative* to fixations during the respective adaptation trial, (*t*(112)=1.73; *p*<0.05). This suggests that, indeed, image location can affect scan paths, but does not account for the renewed interest in the eyes seen in the aligned trials (figure 4; movies in the electronic supplementary material).

## 4. Discussion

### (a) Factors contributing to adaptation and rebound

The ‘renewed interest’ observed in the present study could be the result of several factors. One trivial account of rebound would be that the greater the physical (pixelwise) dissimilarity across images, the larger the rebound. While physical similarity may still account for some aspects of the adaptation phenomenon, our results suggest that, at a minimum, additional processes are involved. The animal pictures were examples of different species (birds) or breeds (dogs), showing considerable variability in shape and colour (figure 1*a*), whereas rhesus face pictures were taken from a population of conspecifics, which are relatively constant in shape and colour. In spite of the visual similarity, monkeys showed *greater* rebound for repetition of conspecific faces than for repetition of animals (figure 2*a*) relative to basic-level and control values. Even more striking, aligned and misaligned composites, which consist of identical visual elements, elicited differential rebound from adaptation; sensitivity to change in the aligned face was greater than that in the misaligned face (figure 3).

One might argue that the visual recognition system normalizes to the variation of perceived objects and, therefore, would need greater variation in bird and dog pictures to elicit the same amount of rebound. This alternative account does not explain the original finding of individuation of various whole monkey images but not within-species animal pictures (Humphrey 1974), nor does it account for the different rebounds observed for marmoset versus macaque faces in the present experiment 2 (figure 2*b*).

A final point of interest is that the monkeys in this study showed higher preference ratios for the animals and marmoset faces than for the macaque faces (figure 2). In previous reports, monkeys have shown asymmetrical preferences for various object categories—including animals (Murai *et al*. 2004)—and equal preferences for some inanimate objects and faces (Pascalis & Bachevalier 1998). In the present study, the inclusion of mirror image or rotated controls as well as basic-level comparisons was critical for teasing apart the preference due to *differentiation* of images from that due to overall interest. Although dogs were apparently quite interesting as a class, two consecutive dogs were not nearly as interesting as a dog that followed a face. In contrast, macaque faces, which were not as interesting overall, showed the opposite pattern: a face was more interesting after viewing another face than after viewing an animal. Taken together, it is not only the physical difference, nor the overall interest, but also what Humphrey (1974) referred to as the *differing significance* between two consecutive pictures that may be responsible for the adaptation effects observed in this study.

### (b) Holistic processing revealed by scan paths

In faces, the eyes are the most salient feature and the most reliable cue for recognition and identification in monkeys (Kyes & Candland 1987; Keating & Keating 1993; Nahm *et al*. 1997; Guo *et al*. 2003; Gothard *et al*. 2004) and humans (Yarbus 1967). In experiment 3, if monkeys are viewing aligned and misaligned stimuli based on individual features, then the only new information in the novel trials comes from the lower half of the face. Decreased fixations to the top eye region are expected, due to adaptation of those features relative to the new lower half features. Likewise, if new aligned bottom halves are ‘noted’ more than new misaligned bottom halves due to their proximity to the eye region, then fixations to the adapted eye region should decrease. Only if faces are processed as a whole, should we see scan paths characteristic of viewing new individuals, namely a renewed interest in viewing the eye region (Guo *et al*. 2003). Whereas fixations to the same eyes continued to drop over the misaligned trials, they were conspicuously preserved in the aligned trials. Paradoxically, change in the *bottom half* of the face induced renewed fixation to the *top half*. The fixations immediately above the altered bottom half of misaligned trials, though ambiguous, would be consistent with attempts to fixate the expected eye location of the new bottom half image (figure 1 in the electronic supplementary material). Moreover, the decrement of eye fixations across misaligned trials could not be explained by the offset of the eye region relative to that of aligned trials. To our knowledge, these observations mark the first demonstration that natural behavioural responses during face viewing are driven by holistic processes.

## 5. Conclusion

Our aim in this study was to shed light on the behavioural hallmarks of face perception in monkeys. We adopted the approach used by developmental psychologists to probe the discrimination abilities of pre-verbal infants using habituation (Cohen & Strauss 1979) or preference (Fantz 1961). Following as closely as possible to a similar procedure designed for monkeys (Humphrey 1974) while testing criteria derived from human psychophysics, we show evidence that monkeys have expertise in face perception.

Like humans, monkeys individuate conspecific faces but not non-face category exemplars such as birds or dogs. This individuation is species specific: the perceived difference of macaque faces exceeds that of marmoset faces. Further, holistic processing was revealed through measurements of rebound from adaptation and scan paths. Alterations in parts of a face caused a renewed interest in the whole face, as though a new individual had been presented. These results suggest that monkeys and humans naturally perceive conspecific faces similarly.

## Figures and Tables

**Figure 1:**
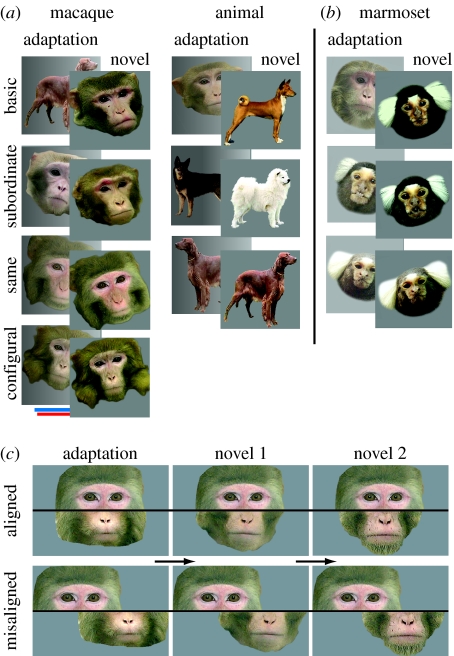
(*a*) Sample stimuli for experiment 1. Trial types included four monkey and three animal conditions, defined by differences between prior (adaptation) and present (novel) images. Trial types overlapped during the task; every novel trial served as an adaptation trial for the next stimulus (except for the last novel stimulus) for a total of seven rebound trials per session. In the configural condition, the inter-ocular distance for the example monkey face is shown for the adaptation trial (blue line) and the novel trial (red line). (*b*) Sample stimuli for experiment 2. Marmoset images replaced the bird or dog images from experiment 1, and new monkey images were used (as in (*a*)). (*c*) In experiment 3, composite images within a condition were presented consecutively, with each novel trial displaying a new bottom half. Note that in each session, different top halves were used for the aligned and misaligned conditions, i.e. the composites shown here were not presented in the same session.

**Figure 2:**
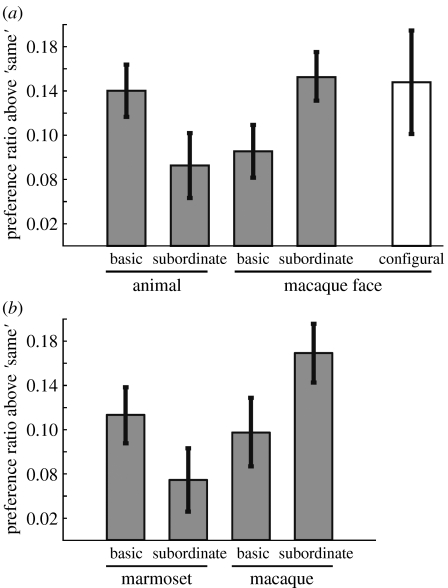
(*a*) Experiment 1: differences in mean preference ratio between each experimental condition (basic, subordinate and configural) and the same condition. Error bars indicate s.e.m. Greater rebound from adaptation was seen for the subordinate level of macaque faces than for either the basic level of faces or the subordinate level of animals. The significant interaction of stimulus category and level shows that faces, but not animals, were perceived at the subordinate level. Rebound for configurally manipulated faces (white bar) also exceeded that of the same condition. (*b*) Experiment 2: as in experiment 1, greater rebound from adaptation was seen for the subordinate level of macaque faces than for the subordinate level of marmoset faces, with a significant interaction between category level and species.

**Figure 3:**
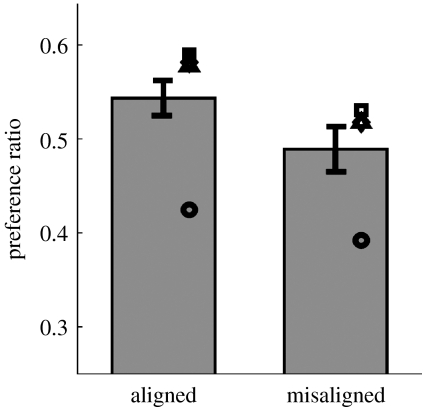
Preference ratio for aligned and misaligned composites (experiment 3). Aligned composites produce greater rebound than misaligned composites, consistent with holistic processing of the aligned stimuli. Symbols reflect the preference ratio for individual monkeys; error bars reflect the s.e.m.

**Figure 4:**
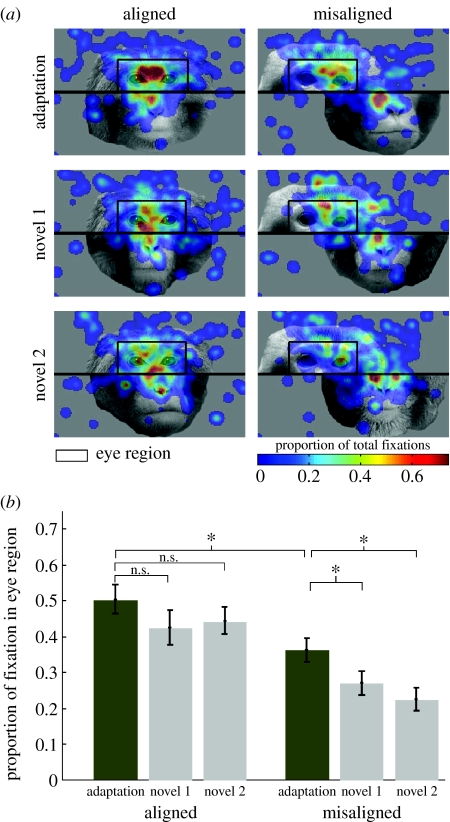
(*a*) Total fixation densities for each of the six trial types in experiment 2. The outline demarcates the eye region used for analysis. For reference, fixation densities are superimposed on greyscale versions of one set of face composites. (*b*) The proportion of fixation time within the eye region to total fixation time on the image, occurring within each 8 s rebound window. Whereas in the aligned condition, the novel trials do not differ significantly from the adaptation trials, the eye fixations in the misaligned novel trials are significantly decreased. Asterisk indicates a significance level of *p*<0.05 (corrected for multiple comparisons).

## Supplementary Material

Fixations in top background The proportion of fixation durations for the upper background region to total fixation duration. For each condition (aligned or misaligned) data were pooled across sessions and trials (Adaptation and Novel). Fixations in the top half of the misaligned condition (*t*(166)=−2.37; *p*<0.01).

Misaligned adaptation scanpath An example scanpath for an original misaligned trial

Misaligned rebound scanpath An example scanpath after a new bottom half is presented

Aligned adaptation scanpath An example scanpath for an original aligned trial

Aligned rebound scanpath An example scanpath after a new bottom half is presented
